# The role of the anterior temporal cortex in action: evidence from fMRI multivariate searchlight analysis during real object grasping

**DOI:** 10.1038/s41598-022-12174-9

**Published:** 2022-06-05

**Authors:** Ethan Knights, Fraser W. Smith, Stéphanie Rossit

**Affiliations:** School of Psychology, University of East Anglia, Norwich, UK

**Keywords:** Neuroscience, Cognitive neuroscience, Motor control, Sensorimotor processing, Sensory processing, Visual system

## Abstract

Intelligent manipulation of handheld tools marks a major discontinuity between humans and our closest ancestors. Here we identified neural representations about how tools are typically manipulated within left anterior temporal cortex, by shifting a searchlight classifier through whole-brain real action fMRI data when participants grasped 3D-printed tools in ways considered typical for use (i.e., by their handle). These neural representations were automatically evocated as task performance did not require semantic processing. In fact, findings from a behavioural motion-capture experiment confirmed that actions with tools (relative to non-tool) incurred additional processing costs, as would be suspected if semantic areas are being automatically engaged. These results substantiate theories of semantic cognition that claim the anterior temporal cortex combines sensorimotor and semantic content for advanced behaviours like tool manipulation.

## Introduction

The human ability to use tools (like using a knife for cutting) symbolises a great step in our evolutionary lineage^1^, but the brain mechanisms underpinning this behaviour remain debated. Over the past decades, theoretical models^2–4^ and neuroimaging evidence converge to propose that intelligent tool-use is the result of functionally interacting neural systems (for recent summaries see^5,6^). One such neural system is the posterior parietal sensorimotor circuit proposed to perform conceptual processing about the objects during sensing and handling by cognitive embodiment theories^7,8,9^. The classic dual visual stream theory^10^ further incorporates visual ventrally located brain areas (e.g., Lateral Occipital Temporal Cortex) for perceiving tool properties (e.g., visual form, shape^11^). Additional dual stream models describe the Inferior Parietal Lobule (IPL) and posterior Middle Temporal Gyrus (MTG) as neural sites that integrate information from sensorimotor and perceptual brain regions into a visuo-kinesthetic format relevant for tool manipulation^12,2,4^. Most recently, focus has shifted toward the role of the anterior temporal cortex in tool-use (e.g.,^13^, based on claims from semantic models that this area constitutes an amodal hub which weaves abstract conceptual representations^14,15^).

Each of these ‘tool-use’ brain regions have been identified by seminal picture-viewing neuroimaging studies (e.g.,^16^). The involvement of posterior/inferior parietal and lateral occipital cortices initially suggested to code tool-related information by picture viewing studies has since been replicated by a small number of functional MRI (fMRI) experiments involving real tool manipulation^17^, ^18,14–21^, see Valyear et al.^22^ for a review. The anterior temporal cortex, however, has yet to be identified with real action tasks during which participants are asked to manipulate tools with their hands. This is at odds with traditional neuropsychology evidence showing that anterior temporal lobe degeneration in semantic dementia patients causes the loss of conceptual knowledge about everyday objects, despite retained shape processing and praxis^23,24^. In fact, converging neuroimaging evidence shows that anterior temporal cortex represents conceptual information about tools, like the usual locations or functions associated with a tool, but these findings are restricted to high-level cognitive tasks thought to rely on mechanisms distinct from real hand-tool manipulation^25,26^, such as picture recognition, language or pantomime^90,22–31^.

**Figure 1:**
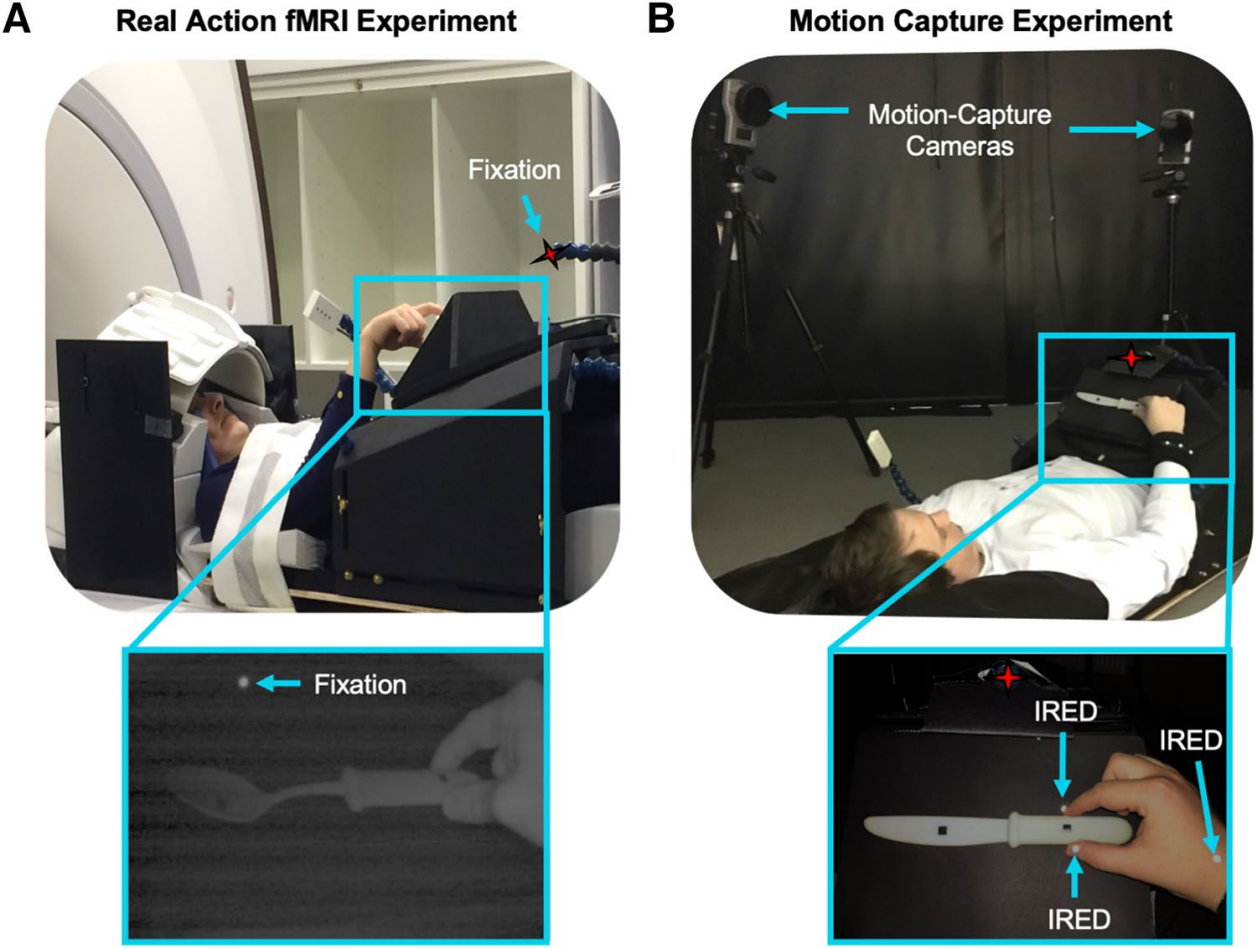
(**A**) *fMRI Experiment*. Participants laid under a custom-built MR-compatible turntable where 3D-printed tool and non-tool stimuli were presented within reaching distance in a block-design. (**B**) *Motion-Capture Experiment*. As a behavioural control experiment, participants performed this paradigm in a motion-capture laboratory to measure kinematics with infrared-reflective (IRED) markers affixed to the hand. (**A** and **B**) During the experiments, the rooms were completely dark, objects were visible only when illuminated, all actions were performed with the right-hand only and participants were naïve to study goals (i.e., they were asked to grasp right or left side of objects without mentioning we were investigating tools or typicality manipulation).

**Figure 2:**
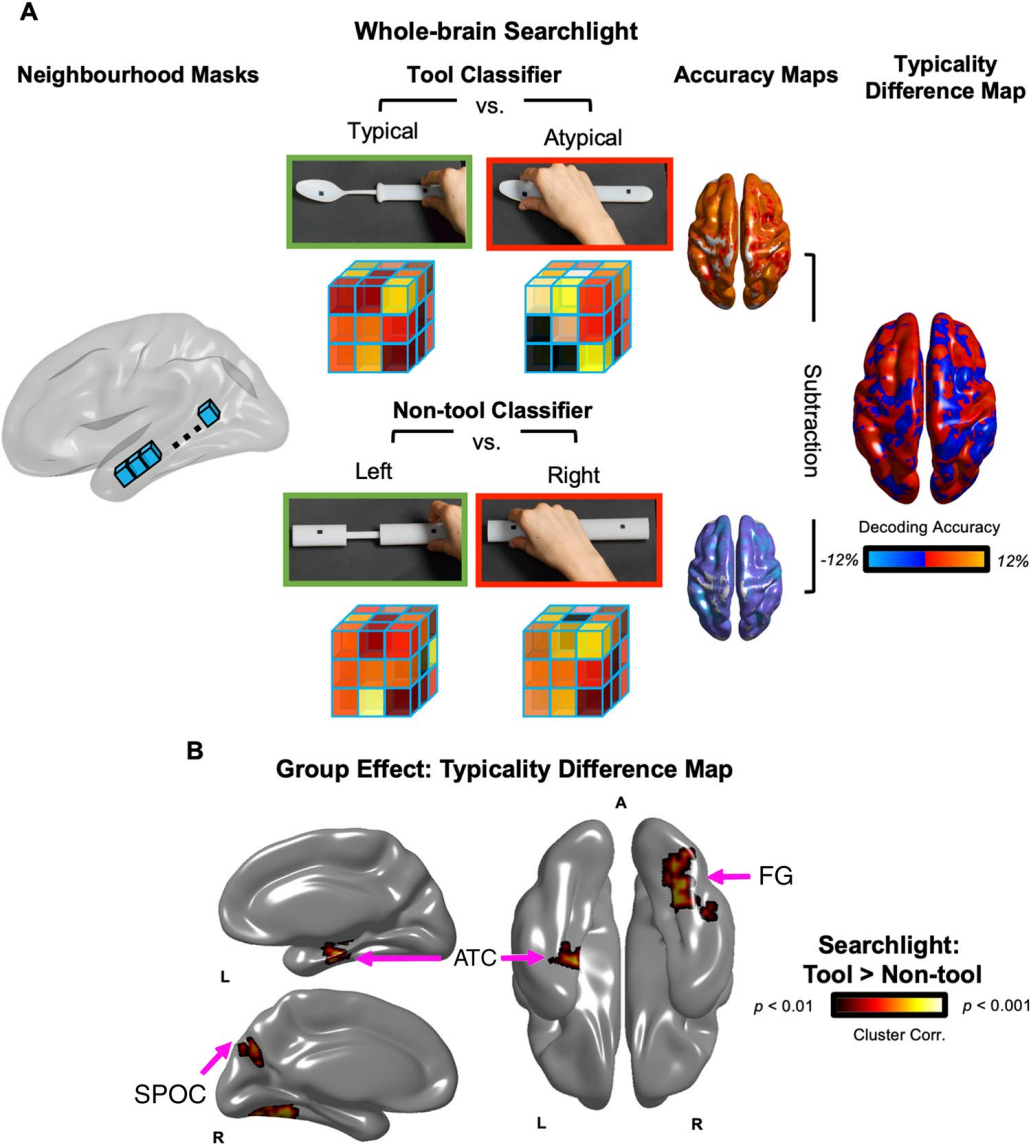
(**A**) *Wholebrain searchlight classification*. For each participant, brain activation patterns were extracted from a mask (single blue cube) that was shifted through the entire fMRI volume. Decoding accuracy was measured with independent linear pattern classifiers for tool (top row) and non-tool actions (bottom row) that were trained to map between brain-activity patterns and the type of grasp being performed with the tools (typical vs. atypical) or non-tools (right vs. left). Typicality difference maps were produced by subtracting the decoding accuracy maps for tools and non-tools, as well as chance-level accuracy (50%). (**B**) *Searchlight Results*. The group typicality difference map demonstrated clusters in the left anterior temporal cortex, as well as right medial parietal and fusiform areas, where decoding accuracies were significantly higher for actions with tools (typical vs. atypical grasps) than non-tools (biomechanically equivalent right vs. left grasps). Acronyms: ATC: Anterior Temporal Cortex; FG: Fusiform Gyrus; SPOC: Superior Parieto-Occipital Cortex.

To test across the whole-brain which regions are sensitive to learned tool-use knowledge, we applied whole-brain searchlights to an fMRI dataset in which participants performed real hand actions with 3D-printed tools. Participants grasped 3D tools in ways that were considered typical for use (grasping a spoon by the handle) or not (grasped by the tool-head; Fig. 2A; re-analysis of Knights et al.^20^). To ensure the decoding effects between typical vs. atypical actions were independent of kinematic differences, we also included biomechanically matched actions with control non-tools (grasping right vs. left). In a control behavioural experiment, we lastly tested if these tool and non-tool actions were appropriately matched for biomechanics by recording hand kinematic using high-resolution motion-capture during the same paradigm outside the MR environment (Fig. 1B).

## Results

### Real action fMRI experiment

Whole-brain searchlight Multivoxel Pattern Analysis (MVPA) (Fig. 2A)^32,33^ was used to identify the brain regions that represented how to appropriately grasp tools for use (i.e., by handle rather than tool-head). Specifically, a stringent typicality difference map (Fig. 2) was generated using a searchlight subtraction analysis that controlled for low-level hand kinematics: the multivariate decoding map of right versus left grasps of control non-tools was subtracted from the decoding map of typical (right) versus atypical (left) grasps of tools (see Methods). This difference map thus reveals which brain areas contain information about how to grasp tools correctly for subsequent use, independently of low-level differences between right versus leftward grasping movements.

As presented in Fig. 2, significantly higher decoding accuracy for tools than non-tools was observed in a large cluster (see Table 1 for cluster sizes) comprising an anterior portion of the left Superior and Middle Temporal Gyri (STG; MTG) that extended into the Parahippocampal Gyrus (PHG). Other clusters surviving correction for multiple comparisons included those within the right Fusiform Gyrus (FG) and anterior Superior Parieto-Occipital Cortex (aSPOC). No cluster of activity demonstrated higher decoding accuracy in the reverse direction, that is, for non-tools higher than tools.

**Table 1:** Searchlight result cluster sizes (voxels), peak coordinates (Talairach) and statistics.

Region	Cluster size	X	Y	Z	t-statistic	*p*
L-MTG	1674	− 39	− 16	− 11	5.6	< .001
L-STG		− 45	− 7	− 5	5	< .001
L-PHG		− 27	− 19	− 23	4.8	< .001
R-FG	1410	30	− 73	− 5	4.8	< .001
R-SPOC	278	15	− 67	31	4.64	< .001

### Behavioural motion-capture experiment

**Figure 3:**
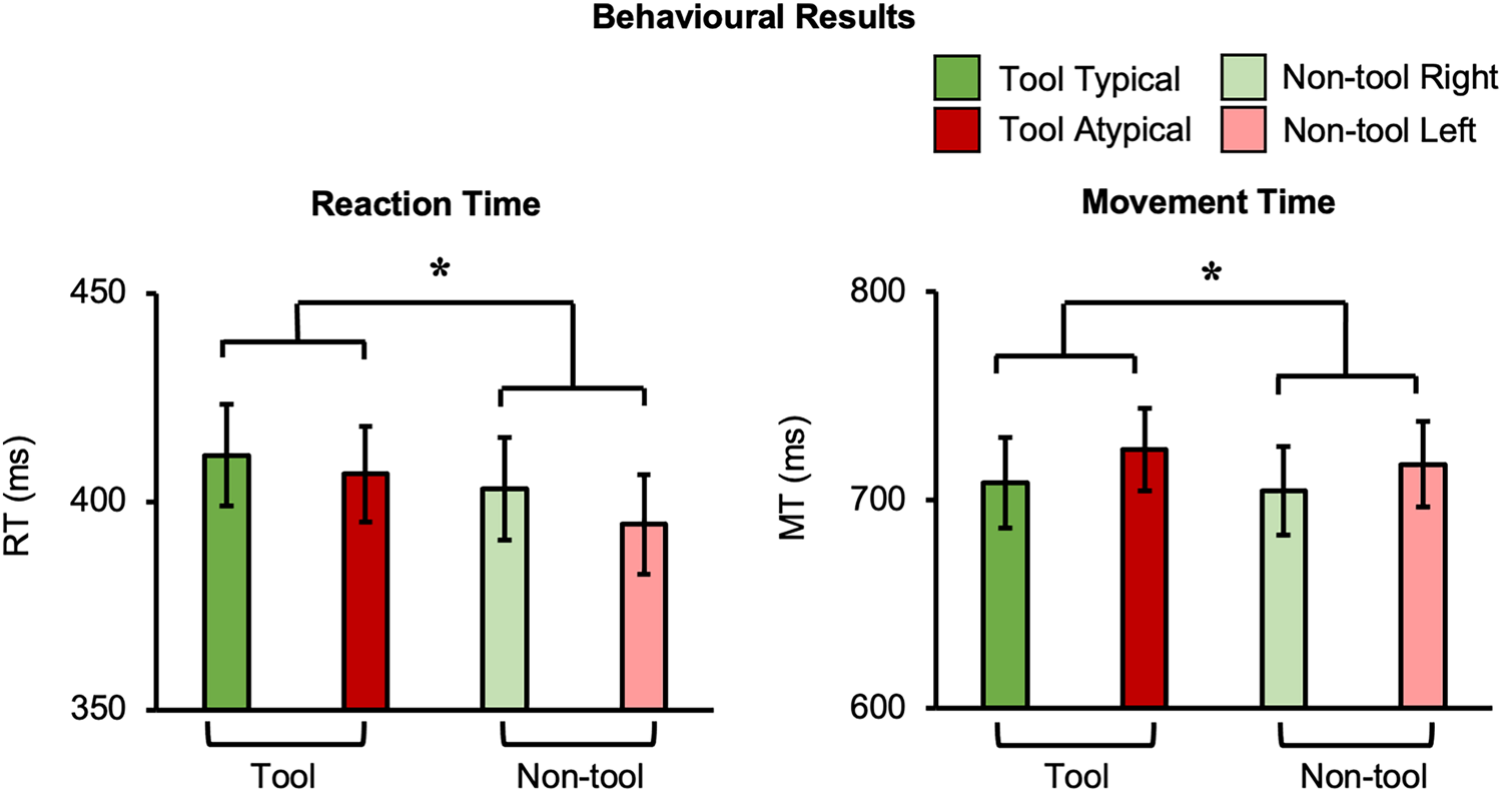
*Behavioural Results*. Hand kinematics differed between object categories: participant’s RTs and MTs were slower when grasping tools, relative to non-tools. Error bars represent standard error of the mean.

To better understand action processing speed for tools vs. non-tools, we measured hand kinematics with high-resolution motion-capture while participants performed the same task outside the MRI. As presented in Fig. 3, analysis of reaction time (RT) and movement time (MT) both revealed a significant main effect of object category (RT: F(1,21) = 15, *p* = 0.001, ηp2 = 0.42; MT: F(1,21) = 5.74, *p* = 0.026, ηp2 = 0.22) where grasping was slower for tools than non-tools (RT mean difference [standard error] = 9.7 ms [2.5 ms]; MT mean difference = 6 ms [2.5 ms]). Overall effects of reach direction (i.e., slower across-body reaches) were also observed where leftward (relative to rightward) actions had longer MTs (F(1,21) = 8.9, *p* = 0.007, ηp2 = 0.3) and a decreased peak velocity (PV) (F(1,21) = 11.48, p = 0.003, ηp2 = 0.35) (MT mean difference = 14.8 ms [5 ms]; PV mean difference = 34.4 ms [10.2 ms]) (Fig. S1). No other significant main effects or any interaction between object category and typicality were found (all *p*’s > 0.15). Importantly, this lack of interaction indicates that timing did not differ specifically for grasping tools typically vs. atypically when compared to the matched movements with control non-tools.

## Discussion

Our real action searchlight analysis presents the first fMRI evidence that left anterior temporal cortex is sensitive to action information about tool movements during real 3D object manipulation (Fig. 2B). These results are in line with recent tool-use models (e.g.,^13^ that include claims from semantic cognitive theories about the role of anterior temporal cortex in constructing abstract object representations^14,15,34^). According to these leading models, the anterior temporal cortex processes conceptual knowledge that is feature invariant (i.e., generalises across exemplar identities) like the typical way tools are handled for use (i.e., grasp tool by its handle), as demonstrated here.

Anatomically, the reported neural region peaks here are near anterior temporal lobe clusters known to code semantics during tool pantomimes^27^ or tool manufacturing^35^. The peak location of these regions is further along the posterior axis than reported during object knowledge association tasks (e.g.,^31^), but general standardised neuropsychological tests of associative knowledge have been reported at comparable locations (e.g., ^36^). Implementing specialised distortion correction ^37^ or an increased field of view^38^ will be useful for future fMRI studies to address whether areas further along the temporal pole also code information about tool manipulation. As for the left lateralisation of this effect, this resembles a popular model of the left hemisphere tool processing networks^3^ and is in line with the fact that all movements were performed with the right-hand during our study. Moreover, left lateralised anterior temporal responses have been reported for semantic language processing^38^ which, when considered alongside our results, resembles the prevalent view across philosophy^39^, and more recently neuroscience (e.g.,^40,41^), that language and motor skills are tightly linked.

Remarkably, this tool-related semantic content was detectable even when task performance was independent to tool conceptual processing. That is, unlike prior tasks that have asked participants to explicitly attend to different tool associations ﻿(﻿e.g., pantomiming actions related to scissors vs. pliers^27^ or recalling if a tool is typically found in the kitchen vs. garage^31^), our participants were simply instructed to grasp the ‘left’ or ‘right’ side of the stimuli and, throughout all aspects of experimentation (see Methods) the stimuli were purposefully referred to as ‘objects’ (rather than ‘tools’). Since participants were not required to form intentions of using these tools, or even process their identities, our results therefore demonstrate that tool representations are automatically triggered. Like similar findings (e.g., ^42,17,43^), this automaticity supports influential affordance theories^2,35–46^ which predict that merely viewing objects potentiates action. Our results provide evidence of this phenomena for humans at a fine spatial resolution (e.g., compared to the Bereitschaftspotential^47^) and during realistic object manipulation (i.e., for tool-use that are directly viewed without the use of mirrors).

Representations about actions with tools also extended into the fusiform and medial parieto-occipital cortex (Fig. 2B), consistent with previous views that these areas code semantics, due to either showing crossmodal responses (e.g., reading tool words and viewing tool pictures^39–50^; or representing learnt object-associations^51^). In fact, our results are in line with the hub-and-spoke theory^52^ suggesting that these two domain-general systems (e.g., for perception or action^10^), may act as spokes to a left anterior temporal cortex ‘hub’ when automatically processing learnt tool movements. Indeed, fusiform cortex is well known for processing perceptual information about object form (e.g.,^53^). And SPOC, along with corresponding regions across the medial wall (V6Av, V6Ad), are known to be involved in planning hand actions (e.g., pointing, reaching and grasping) in both the macaque^54^ and human brain (e.g.,^55–58^). Both the fusiform and SPOC have previously been shown to sensitive to prior experience, such as for processing typical action routines^59,60^ or object functions^61^. Alternatively, these regions could be implicated in networks supporting inference about object properties and their relationship to the laws of physics (e.g.,^4,62,63^), though this account does not necessarily preclude a role of the anterior temporal cortex in the semantic aspects of tool-use.

Consistent with the neural differences observed by contrasting actions with tool and non-tool objects (Fig. 2B), our behavioural motion-capture results similarly demonstrated slower overall responses for grasping tools than non-tools (Fig. 3). From an experimental perspective, the finding of a general object category main effect independent of reach direction indicates that the biomechanics for actions involving the handle and head of the tools were appropriately matched. In other words, basic kinematic differences between different actions cannot simply explain the tool-specific decoding. Considered theoretically, the observed faster non-tool responses are consistent with many accounts describing how tool-related actions are achieved via psychological (e.g.,^52–66^) and neural (e.g.,^2,10,11,67–70^) mechanisms that are distinct from those used for basic motor control. Similar slowing for tools has been observed in simple button-press RT experiments when comparing pictures of tools and of simple shapes^71^ or other object categories (e.g., natural objects; ^72^). As with our findings, these simple RT effects are thought to be caused by the interference from the additional processing of competing (yet task irrelevant) functional associations that are automatically triggered by viewing tools (e.g.,^73,74^).

By virtue of the grasping paradigm used here, our results are unable to capture which brain regions represent real tool-use (like scooping with a spoon). Our grasping paradigm ensured that biomechanical properties of the movements were tightly controlled across conditions (e.g., by specifying grip points), but ongoing work in our laboratory is extending these paradigms to real tool-use with more variable degrees of freedom. Further, additional functional connectivity approaches utilising Dynamic Causal Modelling (DCM) (e.g.,^75^) will be suited to deepen our understanding of the relationship between the anterior temporal cortex and other systems proposed to support tool-use. For example, DCM could be used to determine whether, as predicted by hub-and-spoke theory^15^, left anterior temporal cortex influences ventral visual stream activity in a bidirectional manner. As a final consideration, we expect that the lack of decoding effects in LOTC and IPS (i.e., regions that we identified to be sensitive to typical tool grasping in a previous analysis of this dataset) is due to our searchlight approach. Searchlight decoding relies on the assumption that information is contained within local clusters of voxels at a specific resolution (i.e., of the sphere or cube^76^), and this resolution is reduced by averaging maps across participants, particularly for regions that are anatomically variable (like the IPS). In Knights et al.^20^ we instead used a Region of Interest (ROI) approach to identify the LOTC and IPS at the subject-level and even used a functional localiser to select functionally relevant voxels (e.g., hand-selective) which likely boosts decoding sensitivity. In fact, examining an uncorrected version of the typicality difference map does reveal a cluster in the left IPS consistent with^20^ (see Fig. S2).

Altogether, neural representations were detected for the first time in anterior temporal areas that leading theories of semantic cognition claim to build rich amodal relationships about objects and their uses. By observing the automaticity of these task-irrelevant effects across both behaviour and the brain, our results begin to uncover which, as well as how, specific brain regions have evolved to support efficient tool-use, a defining feature of our species.

## Methods

### fMRI

#### Participants

Nineteen healthy participants (10 male; mean age = 23 +/− 4.2 years; age range, 18–34 years, described in Knights et al.^20^), performed the fMRI real action experiment, with each providing written consent in line with procedures approved by the School of Psychology Ethics Committee at the University of East Anglia.

#### Ethics

The research was carried out according to the Declaration of Helsinki and approved by the Ethics Committee of the School of Psychology at the University of East Anglia. All participants gave informed consent prior to participation.

#### Apparatus and stimuli

The 3D-printed kitchen tool and biomechanically matched non-tool bar objects were adapted from Brandi et al.^19^ (Fig. 1A). As in Knights et al.^20^, the dimensions of each non-tool were matched to one of the tools, such that variability was minimized and kinematic requirements were as similar as possible between different grasps (i.e., left vs. right and small vs. large), including controlling for low-level shape features that can confound tool-effects, like elongation^77^.

An MR-compatible turntable apparatus was custom-built for presenting the 3D objects within reachable space (Fig. 1; also see^20^). Specifically, objects were placed on the turntable that was located above the participant’s pelvis and were only visible when illuminated by a bright white light-emitting diode (LED). To control for eye movements, participants were instructed to fixate a small red LED positioned above objects. Right eye and arm movements were monitored online and recorded using two MR-compatible infrared-sensitive cameras (MRC Systems, Fig. 1 Left) to verify that participants maintained fixation and performed the correct grasping movements. Participants laid the scanner with a head-tilted configuration (~ 20 deg) to allow direct viewing of the workspace and 3D stimuli without the use of mirrors. An upper-arm restraint and industry standard cushioning were used to minimise motion artefacts by ensuring that movements were performed by flexion around the elbow only. Auditory instructions were delivered via earphones (Sensimetrics MRI-Compatible Insert Earphones Model S14).

#### Experimental design

A powerful block-design fMRI paradigm^20^ maximised the contrast-to-noise ratio, to generate reliable estimates of average voxel response patterns, while also improving the detection of blood oxygenation level-dependent (BOLD) signal changes without significant interference from artefacts during overt movement^78^. Briefly, a block began with an auditory instruction (‘Left’ or ‘Right’) and participants grasped the object during 10 s ON-block when the object was briefly illuminated using a right-handed precision grip (i.e., index finger and thumb) along the vertical axis. Throughout experimentation (i.e., consent materials, training instructions) the stimuli were referred to as ‘objects’ such that participants were naïve to the study’s purpose of examining typical versus atypical tool actions.

#### Acquisition

The BOLD fMRI measurements were acquired using a 3 T wide bore GE-750 Discovery MR scanner. To achieve a good signal to noise ratio during the real action fMRI experiment, the posterior half of a 21-channel receive-only coil was tilted and a 16-channel receive-only flex coil was suspended over the anterior–superior part of the skull (see Fig. 1B). A T1-weighted (T1w) anatomical image was acquired with BRAVO sequences, followed by T2*-weighted single-shot gradient Echo-Planer Imaging (EPI) sequences for each block of the real action experiment, using standard parameters for whole-brain coverage (see^20^).

#### Data preprocessing

Preprocessing of the raw functional datasets and ROI definitions were performed using BrainVoyager QX [version 2.8.2] (Brain Innovation, Maastricht, The Netherlands). Anatomical data were transformed to Talairach space and fMRI time series were pre-processed using standard parameters (no smoothing) before being coaligned to an anatomical dataset (see^20^). For each block of interest, and each single run independently, the timeseries were subjected to a general linear model with predictors per condition of interest, as to estimate activity patterns for searchlight MVPA (6 tool and 6 non-tools blocks per run). A small number of runs with movement or eye errors were removed from further analysis (see^20^).

#### Searchlight pattern classification

Searchlight MVPA^32^ was performed independently, per participant, for tool and non-tool trial types using separate linear pattern classifiers (linear support vector machines) that were trained to learn the mapping between a set of brain-activity patterns (β values computed from single blocks of activity) and the type of grasp being performed with the tools (typical vs. atypical) or non-tools (right vs. left). A cube mask (5 × 5 × 5 voxel length, equal to 125 voxels) was shifted through the entire brain volume, applying the classification procedure at each centre voxel^33^ to measure the accuracy that a given cluster of activity patterns could be used to discriminate between the different tool, or non-tool, actions.

To test the performance of our classifiers, decoding accuracy was assessed using an n-fold leave-one-run-out cross-validation procedure; thus, our models were built from n − 1 runs and were tested on the independent nth run (repeated for the n different possible partitions of runs in this scheme; ^79,80^); ^33,80^, before averaging across n iterations to produce a representative decoding accuracy measure per participant and per voxel. Searchlight analysis space was restricted to a common group mask within Talairach space, defined by voxels with a mean BOLD signal > 100 for every participant’s fMRI runs to ensure that all voxels included in searchlight MVPA contained suitable activation. Beta estimates for each voxel were normalized (separately for training and test data) within a range of − 1 to 1 before input to the SVM ^81^, and the linear SVM algorithm was implemented using the default parameters provided in the LibSVM toolbox (C = 1). Pattern classification was performed with a combination of in-house scripts (Smith and Muckli 2010;^33^) implemented in Matlab using the SearchMight toolbox ^82^.

#### Statistical analysis

Voxel accuracies from searchlight MVPA for each participant were converted to unsmoothed statistical maps. To assess where in the brain coded information about typicality, we used a paired samples t-test approach: non-tool accuracy maps were subtracted from the tool accuracy map, producing single participant typicality difference maps (i.e., tool > non-tool) where it was tested, at the group-level, whether the difference in decoding accuracies for tools versus non-tools was greater than zero at each voxel. The BrainVoyager cluster-level statistical threshold estimator^83,84^ was used for cluster correction (voxelwise thresholds were set to *p* = 0.01 and then the cluster-wise thresholds were set to *p* < 0.05 using a Monte Carlo simulation of 1000 iterations), before projecting results on to a standard surface^85^.

### Behavioural control experiment

#### Participants

Twenty-two right-handed (Edinburgh Handedness Questionnaire;^86^) healthy volunteers completed the motion-capture experiment (6 males, 19–29 years of age, mean age = 22.3, SD = 2.4). Ten participants had completed the previous fMRI experiment. All had normal or corrected-to-normal vision, no history of motor, psychiatric or neurological disorders.

#### Ethics

The research was performed in line with the Declaration of Helsinki and approved by the School of Psychology ethics committee at the University of East Anglia. All participants gave informed consent prior to taking part.

#### Apparatus and Stimuli

Stimuli were the same 3D-printed objects used in the fMRI experiment. A Qualisys Oqus (AB, Gothenberg, Sweden) sampling at 179 Hz, measured the position of small passive markers affixed to the participants’ right wrist and the nails of the right index finger and thumb (Fig. 1B). The MR-compatible turntable apparatus was setup in the motion-capture laboratory identically to the fMRI experiment. This included using the same distances between the resting hand and object centre (43 cm) and the centrally aligned red fixation LED (subtending a mean visual angle of ~ 20° from the centre of stimuli), as well as requiring a comparable head tilt (~ 20°). The two minor differences between the MR and motion-capture environments was that for motion-capture there was no arm-strap or eye-monitoring cameras (though participants completed the same pre-experiment training and received verbal reminders between experimental blocks to maintain fixation and to minimise upper arm movements) and the use of noise cancelling headphones (Bose Corporation, USA) to ensure that the sound of stimulus placement did not provide cues about an upcoming trial.

#### Experimental design

Experimental designs were almost identical across the fMRI and behavioural control experiments. The first difference was that the elements critical for modelling the haemodynamic response (baseline periods between trials) during fMRI were omitted in this behavioural experiment. Second, an additional block was collected due to the risk of excluding trials due to marker-occlusion. On average participants completed seven runs (minimum six, maximum seven) totalling 84 experimental trials and 21 repetitions per condition per participant.

#### Data preprocessing

Kinematic data were obtained by localising the x, y and z positions of the markers attached to the index finger, thumb and wrist of the participants’ right hand (Fig. 1B). These 3D positions were filtered using a low-pass Butterworth filter (10 Hz-cut-off, 2nd order). Wrist marker position determined movement on-offset (velocity-based criterion = 50 mm/s) and, in the case that these value was never exceeded, the local minimum of the velocity trace was used as the offset of the outward reaches^87^.

Trial-level reach kinematic dependent variables (Reaction Time, Movement Time, Peak Velocity and time to Peak Velocity; RT; MT; PV; tPV) were computed per the five grasping repetitions and subsequently collapsed. The grand mean, per participant, for the four conditions were retained after removing problematic trials (2.62%) based on the following cases: marker occlusion (2.09%), incorrect object presentation (0.04%) and participant responses that were extremely slow (0.11%; i.e., > 1000 ms) or in the wrong direction (0.38%).

#### Statistical analysis

Repeated measures ANOVAs were used to compare behavioural performance across conditions in a 2 (object category: tools vs. non-tools) × 2 (typicality: typical vs. atypical) factorial design.

## Supplementary Information

Supplementary Information 1.

## Data Availability

The full raw f/MRI dataset is accessible from OpenNEURO (https://openneuro.org/datasets/ds003342/versions/1.0.0). The motion-capture datasets are accessible from the Open Science Framework (https://osf.io/uy3qa/).

## Code availability

Computer code for running the experiments and analysis of the fMRI (https://osf.io/zxnpv) and behavioural datasets are accessible from the Open Science Framework (https://osf.io/uy3qa/).
