# Supplementary material for: The role of the anterior temporal cortex in action: evidence from fMRI multivariate searchlight analysis during real object grasping
Source: Sci Rep. 2022 Jun 5;12:9042. doi: 10.1038/s41598-022-12174-9 (PMC9167815; doi:10.1038/s41598-022-12174-9)
Supplement: Supplementary file 1 — Supplementary Information 1. [file 41598_2022_12174_MOESM1_ESM.docx]

**Supplementary Information**

**
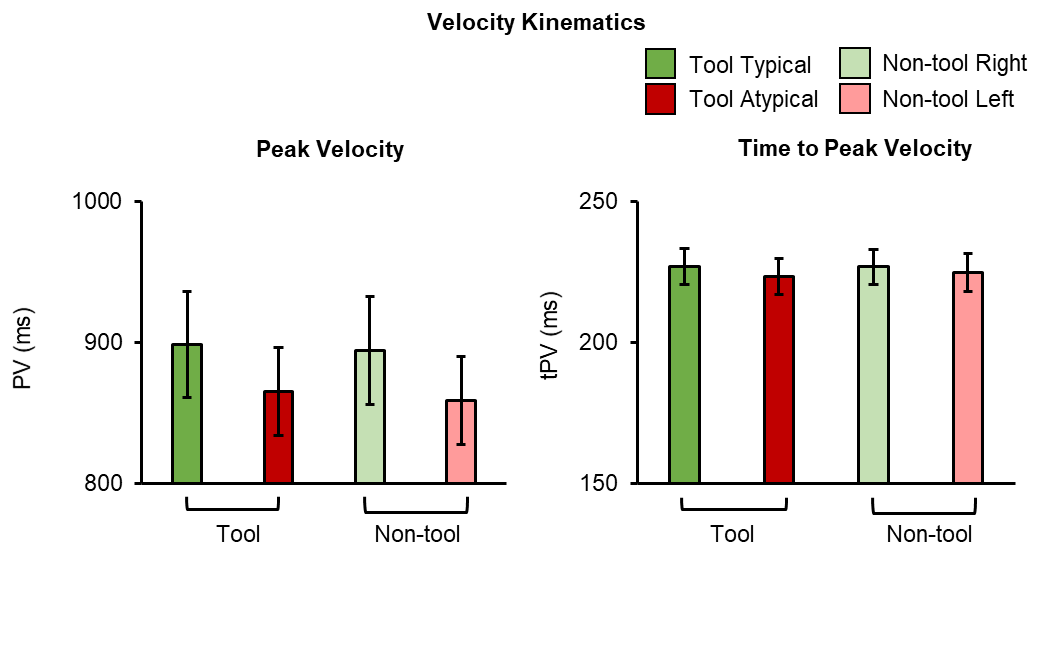
**

Figure S1. **Velocity Behavioural Kinematics.** Like for MT, peak velocity (PV) showed an effect of grasp direction, whereas the time taken to achieve PV (tPV) was consistent across grasping conditions. Error bars represent standard error of the mean.

**
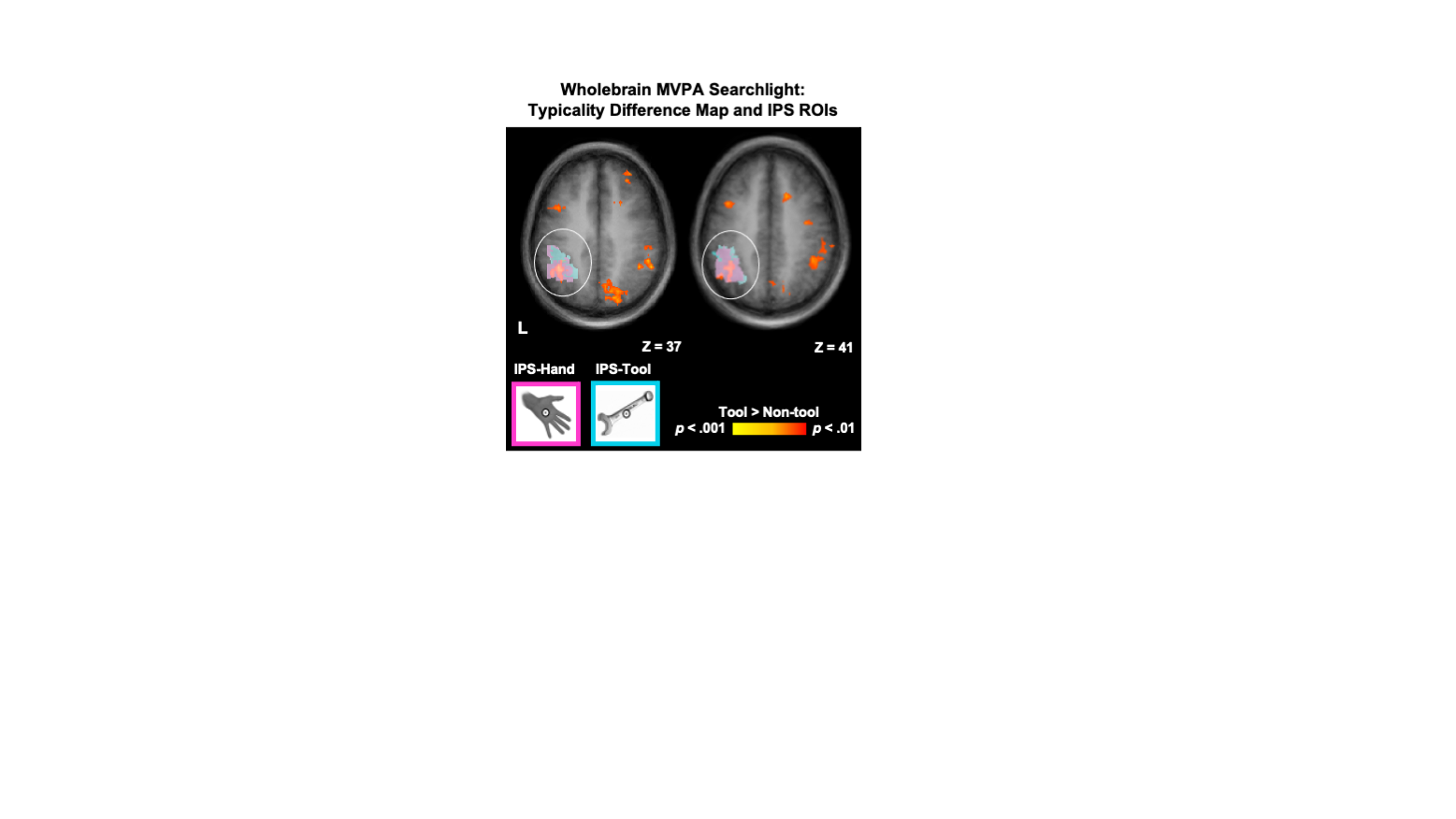
**Figure S2. **Uncorrected Searchlight Typicality Difference Map.** A version of the group typicality difference map without cluster correction shows a cluster in the IPS (circled in white). For reference, the visual functional localiser ROIs from Knights et al. (2021) for hand- and tool-selective IPS (pink & cyan) are overlaid.
